# AI Improves Agreement and Reduces Time for Quantifying Metabolic Tumour Burden in Hodgkin Lymphoma †

**DOI:** 10.3390/hematolrep17060060

**Published:** 2025-11-07

**Authors:** May Sadik, Sally F. Barrington, Johannes Ulén, Olof Enqvist, Elin Trägårdh, Babak Saboury, Anne Lerberg Nielsen, Annika Loft, Jose Luis Loaiza Gongora, Jesus Lopez Urdaneta, Rajender Kumar, Martijn van Essen, Lars Edenbrandt

**Affiliations:** 1 Department of Molecular and Clinical Medicine, Clinical Physiology, Sahlgrenska University Hospital, Sahlgrenska Academy at the University of Gothenburg, 413 90 Gothenburg, Sweden; jesus.lopez.urdaneta@vgregion.se (J.L.U.); martijn.van.essen@vgregion.se (M.v.E.); lars.edenbrandt@gu.se (L.E.); 2 Clinical PET Centre, School of Biomedical Engineering and Imaging Sciences Kings College, London SE5 9RS, UK; sally.barrington@kcl.ac.uk; 3 Eigenvision AB, 211 30 Malmö, Sweden; johannes@eigenvision.se (J.U.); olof.enqvist@gmail.com (O.E.); 4 Department of Electrical Engineering, Chalmers University of Technology, 412 96 Gothenburg, Sweden; 5 Clinical Physiology and Nuclear Medicine, Lund University and Skåne University Hospital, 205 02 Malmö, Sweden; elin.tragardh@med.lu.se; 6 Department of Radiology and Imaging Sciences, Clinical Center, National Institutes of Health, Bethesda, MD 20892, USA; babak.saboury@nih.gov; 7 Department of Nuclear Medicine, Odense University Hospital, 5000 Odense, Denmark; anne.l.nielsen@rsyd.dk; 8 Department of Clinical Physiology, Nuclear Medicine and PET, Centre of Diagnostic Investigations, Rigshospitalet, University of Copenhagen, 2100 Copenhagen, Denmark; annika.loft.jakobsen@regionh.dk; 9 Department of Diagnostic Imaging, Akershus University Hospital, 1478 Oslo, Norway; loaiza_joseluis@hotmail.com; 10 Department of Nuclear Medicine, Post Graduate Institute of Medical Education and Research, Chandigarh 160012, India; drrajender2010@gmail.com

**Keywords:** total metabolic tumour volume, artificial intelligence, Hodgkin disease, observer variation, Fluorodeoxyglucose F18

## Abstract

Background: The aim was to evaluate whether an artificial intelligence (AI)-based tool for the automated quantification of the total metabolic tumour volume (tMTV) in patients with Hodgkin lymphoma (HL) could support nuclear medicine specialists in lesion segmentation and thereby enhance inter-observer agreement. Methods: Forty-eight consecutive patients who underwent staging with [18F]FDG PET/CT were included. Eight invited specialists from different hospitals were asked to manually segment lesions for tMTV calculations in 12 cases without AI advice, and to use automated AI segmentation in a further 12 cases, with editing as required, i.e., segmenting/adjusting 24 cases each. Each case was segmented by two specialists manually and by two different specialists using the AI tool, allowing for the pairwise comparison of inter-observer variability. Results: The median difference between two specialists performing manual tMTV segmentations was 26 cm^3^ (IQR 10–86 cm^3^) corresponding to 23% (IQR 7–50%) of the median tMTV in the dataset, while the median difference between two specialists tMTV adjustments using AI segmentations was 12 cm^3^ (IQR 4–39 cm^3^) corresponding to 9% (IQR 2–21%) (*p* = 0.023). The median difference in tMTV between measurements with and without AI was 3.3 cm^3^, corresponding to 2.3% of the median tMTV. Conclusions: An automated AI-based tool can significantly increase agreement among specialists quantifying tMTV in HL patients staged with [18F]FDG PET/CT, without markedly changing the measurements.

## 1. Introduction

Total metabolic tumour volume (tMTV) and/or total tumour lesion glycolysis (tTLG) has/have been reported to be associated with progression-free and, sometimes, with overall survival in Hodgkin lymphoma (HL) patients staged with [18F]FDG PET/CT [1]. The manual segmentation of every abnormal area of uptake in the [18F]FDG PET/CT is, however, a subjective and time-consuming process, causing inter-observer disagreement. Semi-automated methods based on absolute or relative standardised uptake value (SUV) thresholds have been shown to significantly under- or overestimate visible tumours, thereby limiting the utility of tMTV and tTLG measurements in both clinical practice and clinical trials [2,3]. The calculation of tMTV and tTLG are currently not widely used in clinical practice. We believe an artificial intelligence (AI)-based tool, which has been trained to mimic human readers, has the potential of assisting image readers analysing [18F]FDG PET/CT from HL patients.

A convolutional neural network (CNN) has been developed for non-Hodgkin lymphoma [4], while we developed a tool (RECOMIA) specifically trained on HL patients [5]. As a first step, we compared our tool with a system by PARS (Siemens Medical Solutions USA, Inc.) [6], trained with lesions from lung cancer and lymphoma patients. We found that the RECOMIA and PARS AI tools could be applied without major manual adjustments in 69% (33/48) and 58% (28/48) of patients with Hodgkin lymphoma (HL), respectively.

In this study, we aim to investigate whether the RECOMIA AI-based tool for the automated quantification of the metabolic tumour burden in HL patients staged with [18F]FDG PET/CT could assist nuclear medicine specialists in segmenting focal lesions and thereby improve inter-observer agreement in the quantitative results, and reducing segmentation time.

## 2. Methods

### 2.1. Patients

A total of 49 newly diagnosed, untreated patients with biopsy-proven Hodgkin lymphoma (HL) who underwent staging with [18F]FDG PET/CT at Sahlgrenska University Hospital between 2017 and 2018 were initially included. One patient was excluded due to a failure in recording uptake time. The final cohort comprised 48 patients, with a median age of 35 years (range 7–75), of whom 46% were female. This is the same patient group as reported in previous publications [5,7].

The training of the AI tool is described in [5].

### 2.2. Image Acquisitions

[18F]FDG PET/CT scans were acquired using a Siemens Biograph 64 TruePoint integrated PET/CT system. Patients fasted for at least 6 hours before FDG administration. Adult patients received an injection of 4 MBq/kg [18F]FDG (maximum 400 MBq), while paediatric doses were administered according to the EANM Dosage Card (Version 5.7.2016). The standard uptake time was 60 min. Image acquisition was performed with 3 min per bed position, covering the base of the skull to the mid-thigh. PET images were reconstructed using an iterative OSEM 3D algorithm (4 iterations, 8 subsets) with a matrix size of 168 × 168, slice thickness of 5 mm, and slice spacing of 3 mm. CT-based attenuation and scatter corrections were applied. A low-dose CT scan (64-slice helical, 120 kV, 30 mAs, 512 × 512 matrix) was acquired over the same field of view as the PET scan. CT reconstruction was performed using a filtered back projection algorithm with slice thickness and spacing matched to the PET images [7,8].

### 2.3. Image Interpretation

Eight nuclear medicine specialists (S.F.B., E.T., B.S., A.L.N., A.L., J.L.L., J.L.U., and R.K.) from 8 different hospitals, each with more than five years of experience in interpreting PET/CT studies, were invited to participate. They were asked to segment FDG uptake in tumour sites listed below that should be included in the tMTV and the tTLG (tMTV × SUV_mean_) calculations with and without the AI tool. The specialists were informed that the cohort consisted of untreated HL patients, but no other clinical information such as stage, histology, gender, or age were provided. The following FDG uptake should be segmented as “tumour” based on the recommendations by [1]:

- Viable regions within lymph nodes showing increased FDG uptake;
- Focal FDG uptake in bone marrow or other extranodal sites;
- Focal FDG uptake in the spleen, regardless of splenic size;
- Diffuse splenic uptake exceeding liver uptake (spleen/liver ratio > 1.5 and bone marrow/liver ratio < 1.0), in the absence of reactive bone marrow changes.

The RECOMIA cloud-based software (The AI “Lymphoma FDG-PET/CT segmentation:1.0” can be found at the RECOMIA platform.), was used, providing each examination with PET, CT, fused [18F]FDG PET/CT, and maximum intensity projection images [9]. Interpreters could navigate coronal, sagittal, and transverse planes, and PET images were available in multiple colour scales. By default, images were scaled to an upper SUV threshold of 5, but both the SUV threshold and colour scale could be adjusted based on the reader’s preference. The CT images could be viewed using standard settings, e.g., bone, soft tissue, and lung. The segmentation brush could be adjusted in size and to mark only activity above a defined SUV threshold specified by the user. Before beginning, each specialist received two help videos showing how to perform the analysis and an instruction document explaining the purpose of the study.

Each of the eight specialists analysed 24 cases: 12 cases without the AI tool and 12 other cases with the AI tool (see below). Each case was analysed by four different specialists, two without the AI tool and the other two with the AI tool. The cases were randomly distributed to the specialists. In order to limit learning effects, four of the specialists started with the 12 cases without the AI tool and the other four started with the 12 cases with AI tool. The specialists were asked to record the segmentation time for each case.

Without AI tool: The specialist was asked to manually segment lesions that should be included in the tMTV and tTLG calculations.

**Figure 1:**
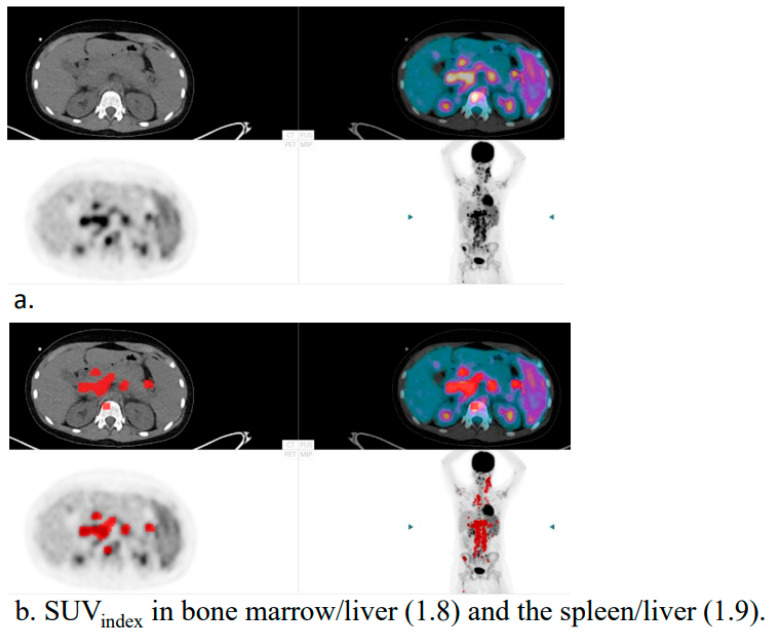
Example of a patient without (**a**) and with (**b**) the automated artificial intelligence (AI) pre-segmentation of tumour burden presented to the specialists (in red). The SUV_index_ in bone marrow/liver and the spleen/liver, calculated by the AI tool, were presented to the specialists (**b**).

With AI tool: The specialist was asked to adjust, as required, the AI lesion segmentations that should be included in the tMTV and tTLG calculations. Focal nodal and extra-nodal lesions detected by the AI tool were highlighted in the PET images (Figure 1). The SUV_index_ in bone marrow (SUV_median_ bone marrow/SUV_median_ liver) and the spleen (SUV_median_ spleen/SUV_median_ liver) were calculated by the AI tool and displayed together with the images (Figure 1). The tool calculated the median values for bone marrow, spleen, and liver. Diffused increased uptake in the spleen that was >1.5 times the liver in the absence of diffusely increased bone marrow uptake was highlighted for the reader in the images (Figure 1). The median SUVs in bone marrow, liver, and spleen were chosen in order to use the most common value and avoid extreme values due to focal lesions [5,8].

### 2.4. AI Tool

The tool, described in detail in [5], is composed of two convolutional neural networks (CNNs), one using only the CT image as input, used to segment tumour in spleen, bone, and liver, and one that uses CT, PET, and an auxiliary mask derived from the CT image as input, designed to directly segment lymph node tumours.

Focal spleen and liver uptake were defined as pixels with SUV above SUVmean + 2SD for that organ. The lymph node CNN uses U-net 3D architecture with two 25% dropout layers. A training set of 101 PET/CT studies from lymphoma patients. Two nuclear medicine specialists performed the segmentation in the training group. A more detailed description of the AI methods can be found in [5].

### 2.5. Statistical Analysis

To evaluate whether the agreement in tMTV measurements had changed using the AI tool, the absolute difference between each pair of specialists who segmented the same cases was calculated. For each image, this gives one difference for values obtained with the AI tool and one without. Kolmogorov–Smirnov and Shapiro–Wilk tests were performed for all analysed parameters showing significant difference (*p* < 0.001); i.e., the data were not normally distributed. Wilcoxon signed-rank test (two-sided) was used to test whether there was a significant difference between these paired measurements. The exact same setup was used to examine the tTLG measurements.

Some readers forgot to record the time for their examinations, but there were 32 cases with timings for both segmentations with and without AI tool. There are several ties since the timings are measured in whole minutes. Therefore, a two-sided sign test was used.

## 3. Results

### 3.1. tMTV: Segmentations with and Without AI Tool

**Figure 2:**
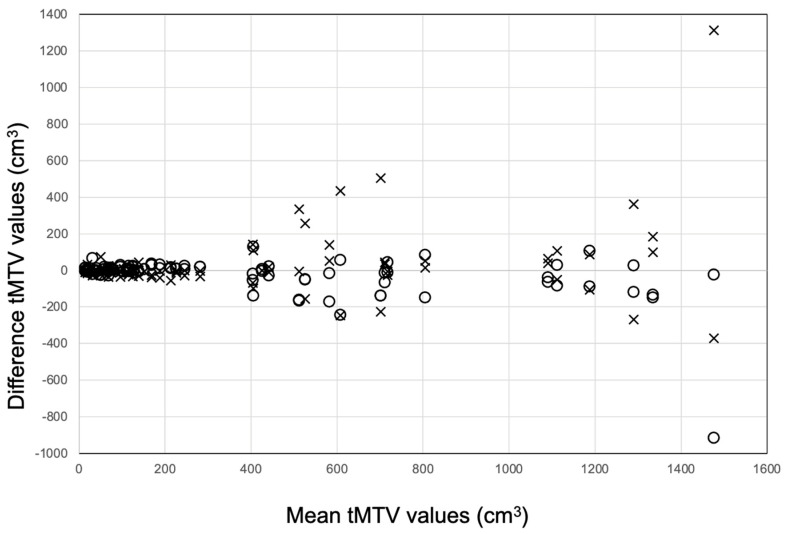
Bland–Altman plot showing the difference between individual specialists’ tMTV values without (X) and with (O) AI support, and the mean of all 4 tMTV values.

**Figure 3:**
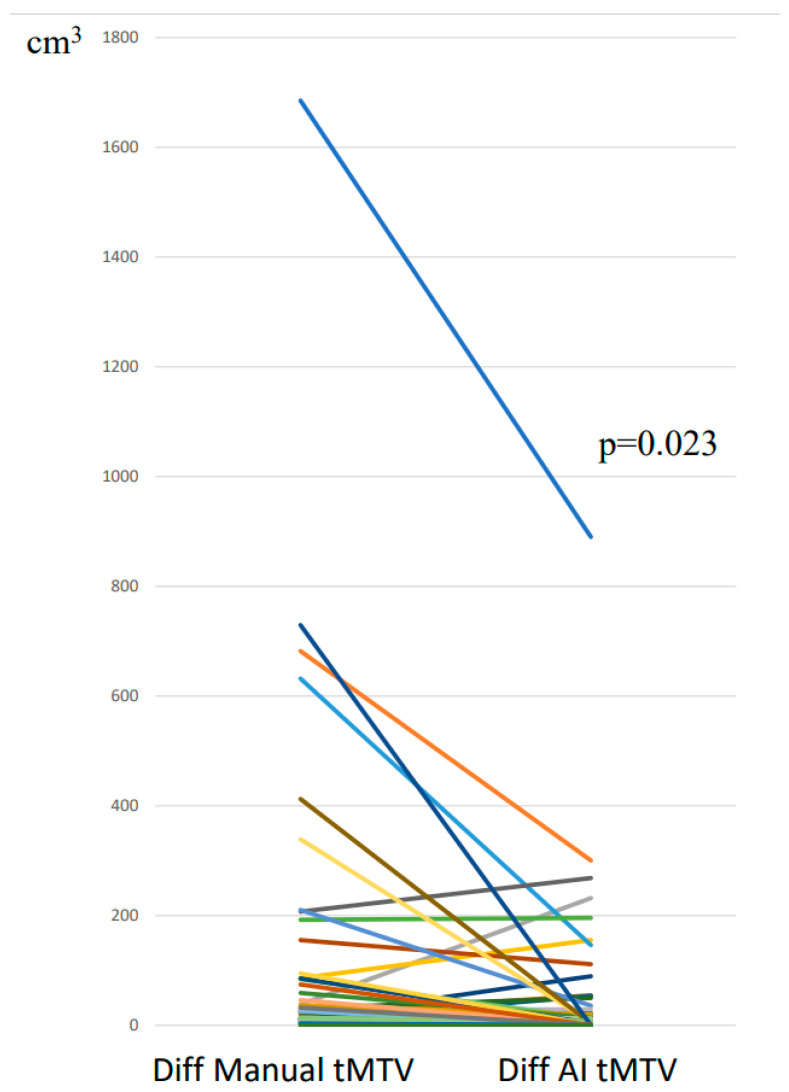
Line graph showing the difference in total metabolic tumour volume (tMTV) between two specialists’ manual segmentations versus adjustments of artificial intelligence (AI) segmentations. Each color line represent the change in each case.

The manual tMTV segmentation ranged between 2–2789 cm^3^, while the specialists’ tMTV segmentation using the AI tool ranged between 10–1451 cm^3^. The median difference between two specialists’ manual tMTV segmentations was 26 cm^3^ (interquartile range (IQR) 10–86 cm^3^) corresponding to 23% (IQR 7–50%) of the median tMTV in the study cohort, while the median difference between two specialists’ tMTV adjustments of AI segmentations was 12 cm^3^ (IQR 4–39 cm^3^) corresponding to 9% (IQR 2–21%). The Bland–Altman plot shows the manual and AI-supported tMTV values (Figure 2). The line graph in Figure 3 indicates significantly less variability in tMTV between two specialists’ segmentations when using the AI tool compared with manual segmentations (*p* = 0.023). The median difference in tMTV between measurements with and without AI was 3.3 cm^3^ corresponding to 2.3% of the median tMTV. In 31 (65%) of the 48 cases, the absolute difference in tMTV between two specialists were lower using the AI tool: in one case, no difference was found with and without the AI tool, and, in 16 (33%) cases, the difference was higher with the AI tool (Figure 3).

### 3.2. tTLG: Segmentations with and Without AI-Tool

**Figure 4:**
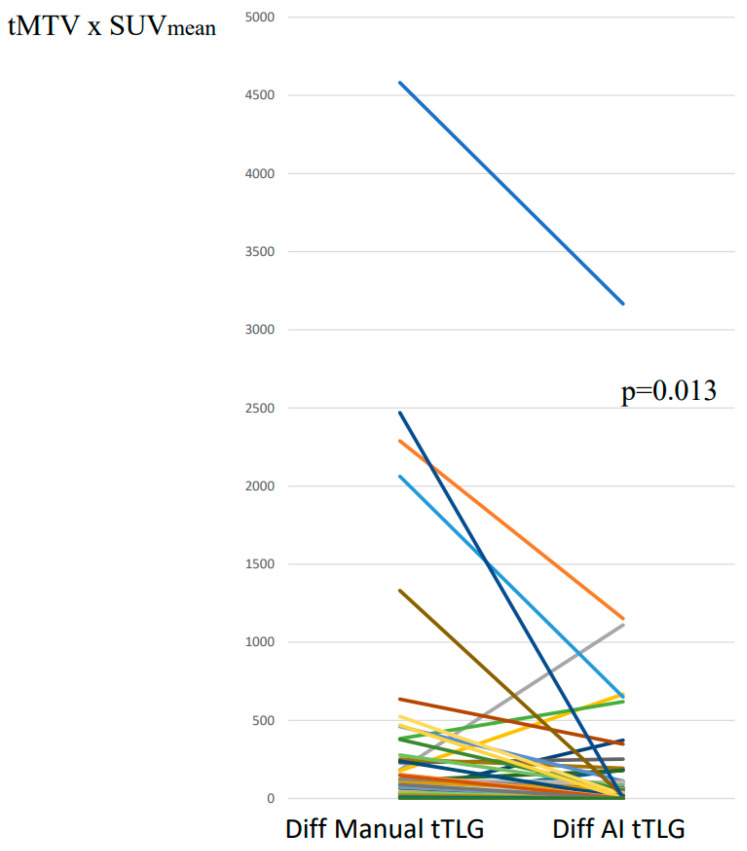
Line graph showing the difference in total tumour lesion glycolysis (tTLG) between two specialists’ manual segmentations versus adjustments of artificial intelligence (AI) segmentations. Each color line represent the change in each case.

The median difference between two specialists’ manual tTLG segmentations was 89 (IQR 29–259) corresponding to 14% (IQR 4–32%), while the median difference between two specialists’ tTLG adjustments of the AI segmentations was 45 (IQR 8–128) corresponding to 6% (IQR 1–13%). The line graph in Figure 4 indicates significantly less variability in tTLG between two specialists’ segmentations when using the AI tool compared with manual segmentations (*p* = 0.013). The median difference in tTLG between measurements with and without AI was 0.1 corresponding to 0.0% of the median tTLG.

### 3.3. Time Registration

Only four specialists reported the time for each case, as intended. In 32 of the 48 cases, at least one time report with and one time report without the AI tool were available. In cases with two time reports for the same method, one of the time reports was randomly selected.

**Figure 5:**
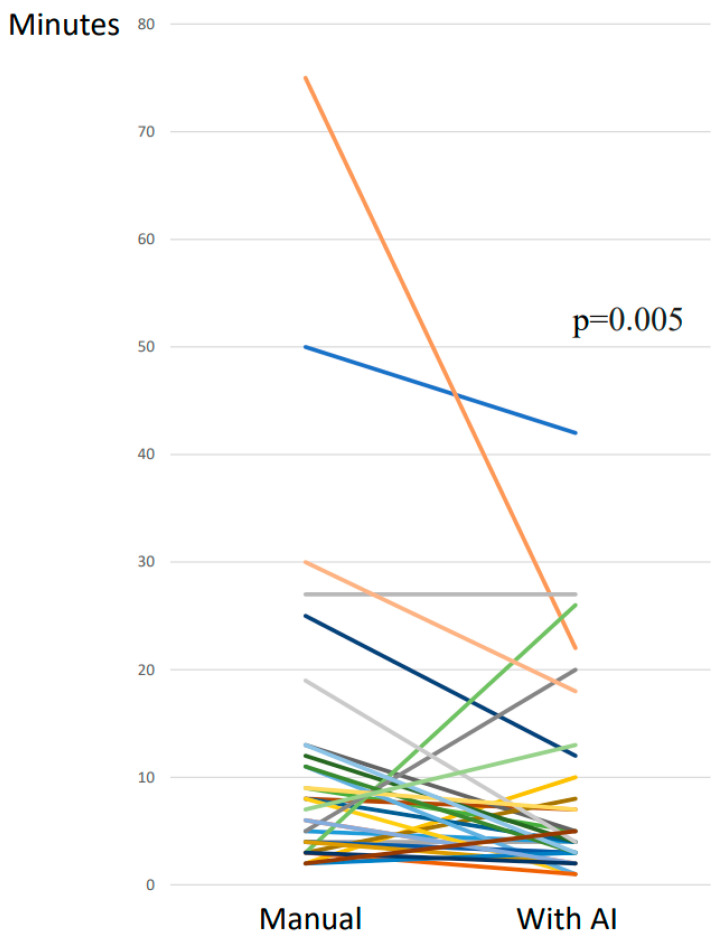
Line graph showing time taken for manual versus artificial intelligence (AI) segmentations. Each color line represent the change in each case.

The median time taken for the manual segmentations was 7.5 min (IQR 4–12), while the median time taken for the adjustments of the AI segmentations was 4 min (IQR 3–10.5). The median difference between the time taken for the adjustment of the AI segmentations versus manual segmentations decreased significantly (*p* = 0.005) by 2 min (IQR 0–8) (Figure 5). In 23 (72%) of the 32 cases, the time deceased using AI compared with manual segmentations; in 2 (6%) cases, the time required was the same, while, in 7 (22%) cases, the time increased using AI.

## 4. Discussion

The present study demonstrated that an AI-based-tool for the automated quantification of the total metabolic tumour burden (tMTV and tTLG) in untreated HL patients staged with [^18^F]FDG PET/CT could assist nuclear medicine specialists in segmenting lesions and significantly improve inter-observer agreement (*p* = 0.023 (for tMTV) and *p* = 0.013 (for tTLG)).

The problem with large deviations in the tMTV between different readers was highlighted in a recent study by Boellaard et. al. in which 10 readers from different hospitals analysed five PET/CT studies from patients with B-cell lymphoma [10]. Despite giving instructions on how to segment the lesions, the authors found large deviations in a first round, because some readers did not follow the instructions but rather acted as they normally do in everyday clinical work. The deviations reported in the first round could indicate how large the tMTV variations are in the clinical routine currently. The authors concluded that there is an urgent need to improve tMTV segmentation workflows in clinical practice. The results in the present study indicate that AI could play an important role in increasing the agreement in tMTV values in clinical practice and research trials.

The median time taken for the manual segmentations was 7.5 min and it decreased significantly when using the AI tool (Figure 5). The analysis time for tMTV segmentations without AI support was reported to be similar in the study of Boellaard et. al. [10], despite the fact that the readers in their study used the software they were used to from their clinical work, while, in this study, they used the RECOMIA platform.

It was not within the scope of the present study to assess the accuracy of the tMTV values. However, the change in tMTV values with and without AI was only 3.3 cm^3^ corresponding to 2.3% of the median tMTV, indicating that this change is not clinically important. As no gold standard exists to validate specialist segmentations due to the unknown ground truth, we compared the tMTV and tTLG measurements obtained by eight experts with each other. A similar approach was used in a study by Boellaard et al. who presented a benchmark method as an alternative to a gold standard. tMTV was measured by 12 PET/CT lymphoma experts in order to establish a reference value for each of the 60 PET/CT studies from lymphoma patients [11]. A strength of our study is that we included eight specialists from eight different hospitals around the world which makes the results generalizable. Both their and our study aim to contribute in different ways to allow tMTV to be a widely used reproducible biomarker.

Several reports have demonstrated that the baseline metabolic tumour burden on [18F]FDG PET/CT has prognostic potential in lymphoma [12,13,14]; however, no consensus exists on how to measure tMTV and tTLG [15,16,17]. Semi-automated methods based on absolute or relative SUV thresholds have been reported to frequently under- or overestimate visible tumours [1,3]. Therefore, we developed a novel approach using AI, trained to mimic human readers, avoiding a specific threshold method.

Despite attempts to validate former threshold methods by phantom studies, no cut-off boundaries have yet been agreed upon to discriminate a good from bad prognosis, nor a cut-off value that justifies a more aggressive treatment regimen [13]. To answer these questions, large cohorts of patients with long follow-up period are needed. We instructed the readers to segment the FDG uptake based on the recommendations by Barrington et. al. [1]. No SUV threshold boundaries were recommended, nor minimum lesion size. This approach mimics the everyday clinical work, which is the intention of this study, rather than to present results from a more artificially arranged study setting.

We aimed to develop an AI tool specifically designed for HL patients and trained the tool using lesions from patients with HL, outlined by two nuclear medicine specialists. To our knowledge, no such AI tool has specifically been developed for adult HL patients, meaning a comparison with other published reports is difficult. Weisman et. al. has developed an AI tool for paediatric HL patients and showed excellent agreement with manual segmentations carried out by specialists both for tMTV and tTLG [18].

Our test set comprised 48 untreated HL patients and eight physicians from different hospitals analysing 24 cases each. These sample sizes were not based on formal power calculations, but experience of what is feasible. The design is similar to the studies by Boellaard et al., who included 10 readers analysing 5 cases each and 12 readers analysing 20 cases each, respectively [10,11].

The segmentation of the total tumour burden is time-consuming and currently not performed in everyday clinical work. We have showed that using AI could significantly reduce the segmentation time and hope that this could increase the feasibility and willingness of reporters to quantify tMTV and tTLG in the daily routine. Our results should be interpreted with caution since only half of the physicians remembered to register the time taken for each case.

Patients were included from a single institution, who had undergone staging for [18F]FDG PET/CT using the same PET/CT system. Interestingly, this small training group could achieve significant improvements, which is promising and suggests that the model has learned robust features even from a limited set. Future work will focus on increasing the training cohort with patients from different hospitals, examined using different cameras to show more lesion varieties to the AI tool. This improved AI tool will also be validated using the standardised tMTV benchmark dataset created by Boellaard and co-workers [11].

In a previous work, we have described the training and testing of the RECOMIA AI tool used here [5]. The current work investigated whether the RECOMIA tool can increase the agreement between specialists quantifying the baseline metabolic tumour burden and decrease the time needed for that. In both papers, the same patient group are used.

The limitations include the fact that the specialists were not familiar with the software used in this study; however, the cases were randomly distributed to the readers, with some starting with manual segmentations while others started with the adjustment of AI segmentations where required. The pairwise comparison of the segmentations, both without and with AI, was made without the knowledge of which segmentation procedure was performed first. Another limitation might be that the comparator with manual segmentation may not be entirely fair, since most commercial software includes tools that facilitate segmentation, such as region-growing algorithms that propagate contours across slices or automatic contouring based on SUV thresholds. However, the manual tool could be adjusted only to segment voxels with SUV values above a specific threshold. Furthermore, no statical adjustments were applied for the three variables investigated (tMTV, tTLG, and time).

The scope of this study was not to test the impact of an AI tool ready for clinical use—to develop such a tool requires a step-by-step huge effort to be taken, including pre-analytical, analytical, and clinical validation of the final product [19,20,21]—nor was the intention to investigate clinical significance of the median reduction of the inter-observer variability of tMTV and tTLG. Large prospective trials are needed for the latter. Furthermore, the test set of 48 patients is too small for a sub-analysis. At present, we are in the early phases of tool development for PET/CT in HL patients. The aim of this project was to investigate if the current tool could improve inter-observer agreement in the quantitative results and reduce the segmentation time and we showed that this could be accomplished despite that the AI tool being based on a relatively small training set, manually annotated by two nuclear medicine specialists. Moreover, the patient examinations used in this study cannot be publicly shared due to ethical considerations. However, the AI tool can freely be accessed on recomia.org.

## 5. Conclusions

An automated AI-based-tool can significantly reduce the segmentation time and improve agreement among specialists quantifying the metabolic tumour burden in HL patients staged with [18F]FDG PET/CT, without markedly altering the absolute tMTV and tTLG measurements. Future work will aim at increasing the training group in order to present more variations to the network and test the tool with an external patient cohort.

## Data Availability

The datasets generated and/or analysed during this study are not publicly available due to ethical restrictions.
